# Time series reconstructing using calibrated reservoir computing

**DOI:** 10.1038/s41598-022-20331-3

**Published:** 2022-09-29

**Authors:** Yeyuge Chen, Yu Qian, Xiaohua Cui

**Affiliations:** 1 School of Systems Science, Beijing Normal University, Beijing, 100875 China; 2 Nonlinear Research Institute, Baoji University of Arts and Sciences, Baoji, 721007 China

**Keywords:** Nonlinear phenomena, Applied mathematics, Computational science

## Abstract

Reservoir computing, a new method of machine learning, has recently been used to predict the state evolution of various chaotic dynamic systems. It has significant advantages in terms of training cost and adjusted parameters; however, the prediction length is limited. For classic reservoir computing, the prediction length can only reach five to six Lyapunov times. Here, we modified the method of reservoir computing by adding feedback, continuous or discrete, to “calibrate” the input of the reservoir and then reconstruct the entire dynamic systems. The reconstruction length appreciably increased and the training length obviously decreased. The reconstructing of dynamical systems is studied in detail under this method. The reconstruction can be significantly improved both in length and accuracy. Additionally, we summarized the effect of different kinds of input feedback. The more it interacts with others in dynamical equations, the better the reconstructions. Nonlinear terms can reveal more information than linear terms once the interaction terms are equal. This method has proven effective via several classical chaotic systems. It can be superior to traditional reservoir computing in reconstruction, provides new hints in computing promotion, and may be used in some real applications.

## Introduction

In recent years, building model-free methods to predict the state evolution of nonlinear dynamic systems with machine learning methods has received increasing attention^1–3^. Among these methods, reservoir computing (RC)^4,5^, a simplified recurrent neural network, has usually played a core role in prediction in many interdisciplinary fields^6–9^. For example, some work investigated the dependence of computing performance on system parameters by time-delay autonomous Boolean node reservoir computing^10^. The Lyapunov exponents^11,12^ of the dynamical system can be estimated from the time series, in addition to the critical transition^13,14^ and the sensing phase coherence^15^. The application of reservoir computing in complex systems and nonlinear dynamics has developed rapidly. Such studies have focused on the identification of chaotic signals^16,17^, inference of partial variables^18,19^, and dynamic observation of excitable systems^20^. In real life, reservoir computing has been applied to some high-dimensional systems, such as the prediction of depletion-induced seismicity^21^, forecasting of atmospheric^22^, control of mechanical sensors^23^, and the fast response of chemosensors^24^.

Among the most prominent examples one applied RC is the prediction of chaotic systems. Classical reservoir computing has achieved 5–6 Lyapunov times in the prediction of large-scale spatiotemporal chaotic sequences^25^. Furthermore, a hybrid forecasting scheme that consists of both reservoir computing and a knowledge model extends the prediction length to 12 Lyapunov times^26^. Other studies proposed to extend the prediction length, such as a framework that uses a special equation to “update” the input information in the prediction phase to obtain long-term effective predictions^27^. It is believed that even a small amount “update” can help various chaotic systems reach an arbitrarily long prediction length. But the equation parameter *c* is not clear. A recent study^18^ used RC with continuous partial variables of the system to infer other unknown variables by changing the model structure. This shows that RC is a very effective and versatile tool for robustly reconstructing unmeasured dynamical system variables. However, it cannot make effective reconstructions when the system variable is symmetric. These studies all demonstrate the practicality of RC in the prediction of chaotic systems, although the prediction length or effect is limited due to the initial sensitivity or some system interaction structures.

In practice, a system may have many variables interacting with each other. However, the observation and recording of all variables in the entire process are impossible or costly. The effective prediction of a variable will benefit our understanding and judgment. Here, we consider a system that has a dynamical model, but the model is not sufficiently accurate. The all variables of the system can be recorded for a short period, and one or two of the variables can be continually or discretely measured. The short period of all variables is used as the “training” set, and the other information is used as the “calibration” in the reconstruction phase. We observed that the reconstruction length ($T_{test}$) can be significantly improved and the reconstruction error decreased. We verified our method by using different time series from several dynamical systems such as the Rössler system, the Lorenz system, the Rucklidge system, the coupled Lorenz system, and so on. The efficiency of reconstruction can be maintained. Moreover, we found that the reconstruction is related to the dynamical interactions. The types and times of interaction between the variables used to “calibrate” and the reconstructed variables will bring different reconstruction.

## Model

We consider a dynamical system $d\mathbf{x} /dt=f(\mathbf{x} )$ with vector valued variables $\mathbf{x} (x_{1},x_{2},\ldots ,x_{n})$. Suppose $x_{i}(i=1,\ldots ,N)$ can be measured over a specific period[0,$T_{train}$], but only some $x_{i}$ can be measured after that period (t >$T_{train}$). We try to replace the trajectories of the variable $x_{i}$ of the same dimension in output variable $\mathbf{s}$ in t >$T_{train}$ only depending on measurable data instead of reconstructing function *f*. Therefore, we use “reservoir computing” which was proposed for the reconstruction of time series, to seek the results.

**Figure 1:**
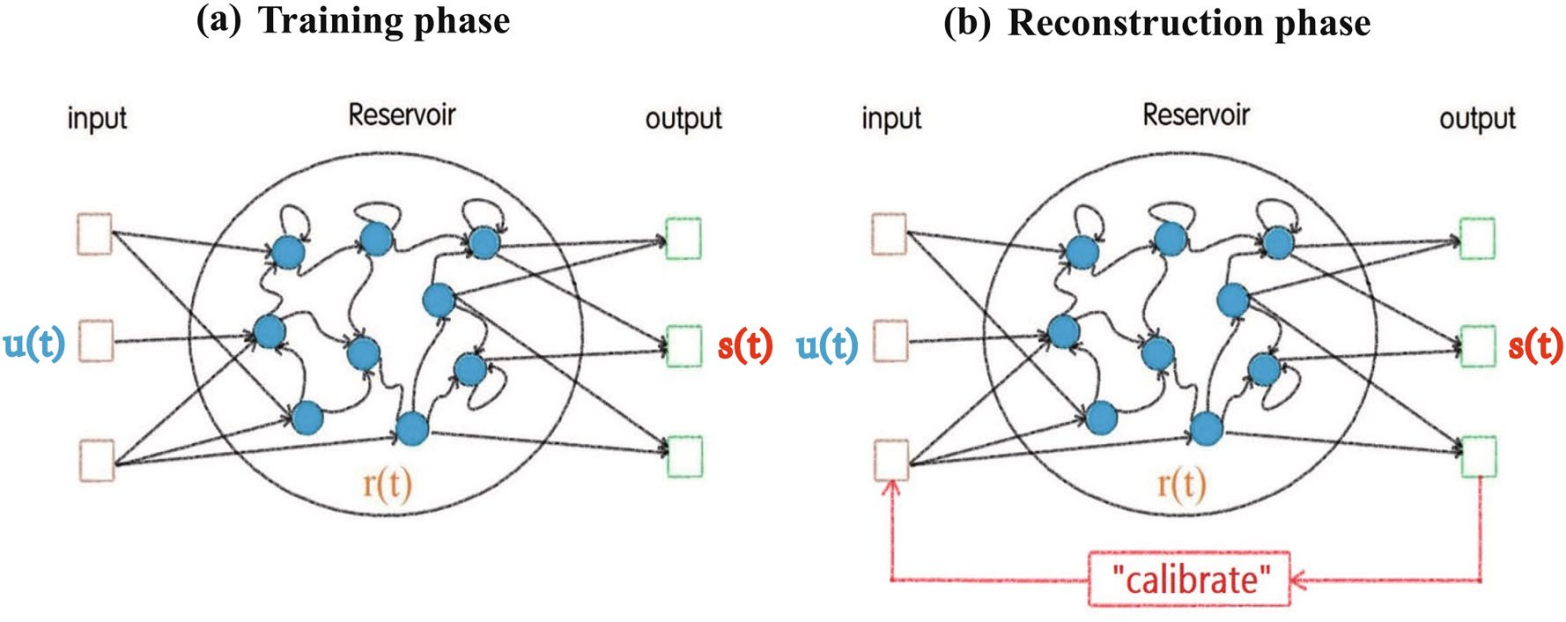
The calculation of the “calibrated” reservoir computing consists of the training phase and the reconstruction phase. (**a**) In the training phase, the input $\mathbf{u} (t)$ and the output $\mathbf{s} (t)$ are known, then the $W_{out}$ is calculated. (**b**) In the reconstruction phase, the biggest difference between our model and the classical reservoir computing is that the output $\mathbf{s} (t)$ is calibrated by the partial actual variables to reconstruct the unmeasured variables accurately.

In this paper, we adopt the reservoir technique proposed by Jaeger and Haas^4^. Reservoir computing is mainly composed of a linear input layer with *M* nodes, a recursive nonlinear reservoir layer with *N* dynamic nodes, and a linear output layer with *M* nodes. We assume that all variables of the system can be recorded in a short period, and partial variables can be continuously recorded over a long period. The variables that can be recorded in the entire process are labeled as measured variables, while the variables that cannot be recorded are labeled as reconstructed variables. Then, we use the measured variable to build a data-driven model to reconstruct the reconstructed variables of chaotic systems and compare the efficiency of reconstruction under different conditions. The solution’s specific content is as follows: use all variables that have been recorded in a short period to train the reservoir, and then use the measured variables to “calibrate” the input for reconstructing the reconstructed variables. Generally, calculation of the model is divided into a training phase and a reconstruction phase, as shown in Fig. 1. Here, the time series data is taken from different three-dimensional systems by numerical calculation.

### Training phase

Compared with other artificial neural network models such as RNN, LSTM, and deep learning^28–30^, the reservoir has far fewer adjustable parameters during the training phase. In our work, we select a standard discrete-time leaky tanh network, so the states of each node of the reservoir update themselves. This obeys the following function:

1 $$\begin{aligned} \mathbf{r} (t+\Delta t)= (1-\alpha )\mathbf{r} (t) +\alpha \tanh (\mathbf{A {} \mathbf{r} (t)+\mathbf{W} _{in}(\mathbf{u} (t+\Delta t)+\xi })), \end{aligned}$$

where $\mathbf{W} _{in}$ is the weight matrix between the input layer and the reservoir layer, the dimension is $N \times (M+1)$. $\mathbf{A}$ is the weighted adjacency matrix of the reservoir layer, the dimension is $N \times N$. $\mathbf{r} (t)$ is the state of each node in the reservoir layer, $\mathbf{r} \in \mathbb {R}^N$ and $\mathbf{u} (t)$ is the *M*-dimensional input vector. *tanh* is the hyperbolic tangent function, which mainly makes nonlinear changes to the input. $\alpha$ is the update speed of each node in the reservoir layer, and $\xi$ is the bias parameter.

In the training phase, the matrix $\mathbf{A}$ and the matrix $\mathbf{W} _{in}$ are randomly selected, and only the output weight $\mathbf{W} _{out}$ needs to be adjusted. Once the input weight $\mathbf{W} _{in}$ and reservoir layer $\mathbf{A}$ are determined, they remain the same throughout the entire process. Therefore, all parameters which are based on them are hyperparameters^31^. Some approaches move the nonlinearity from the reservoir to the output layer^32^, the output layer is chosen to have a linear function to a matrix $\mathbf{R} (t)$ in this paper. Here, we build the matrix $\mathbf{R} (t)=[\mathbf{r} (t);\mathbf{u} (t);\xi ]$ which consists of the reservoir states $\mathbf{r} (t)$, the input $\mathbf{u} (t)$ and bias parameter $\xi$. The output $\mathbf{s} (t)$ at time *t* is described by

2 $$\begin{aligned} \mathbf{s} (t)=\mathbf{W} _{out}{} \mathbf{R} (t), \end{aligned}$$

where $\mathbf{W} _{out}$ is the output weight, the dimension is $M \times (M+N+1)$. During the training phase, the system is open as it requires the output $\mathbf{s} (t)$ from the actual values. The output weight $\mathbf{W} _{out}$ is obtained by matching the output to the actual values in a least-square sense using ridge regression^33^ so that $\mathbf{W} _{out}$ resembles

3 $$\begin{aligned} \mathbf{W} _{out}=\mathbf{s} {} \mathbf{R} ^{T}(\mathbf{RR} ^{T}+\eta \mathbf{I} ), \end{aligned}$$

where, $\eta =1\times 10^{-8}$ is the deviation parameter to prevent overfitting of $\mathbf{W} _{out}$. After the training is completed, we can obtain the output weight matrix $\mathbf{W} _{out}$.

### Reconstruction phase

In classic reservoir computing, after inputting the initial value $\mathbf{u} (t)$, the output $\mathbf{s} (t)$ is used as the input to the closed system. During this phase, the classic method is prone to exponential divergence between the predicted and the actual trajectories, resulting in a short prediction length for chaotic systems. To avoid the divergence, we directly adopt partial actual values to replace the corresponding dimension values of output $\mathbf{s} (t)$, which is different from the synchronization principle^27^. Specifically, we “calibrate” the input in the loop: from the output layer to the input layer, as shown in Fig. 1b. In our “calibrated” reservoir computing, the corresponding dimension of $\mathbf{s} (t)$ is replaced by the measured variables that have been continuously recorded over a long time to obtain a new $\mathbf{s} '(t)$. Taking the Lorenz system as an example, the input variable $\mathbf{u} (t)$ is composed of [*x*(*t*), *y*(*t*), *z*(*t*)]. If *x*(*t*), *y*(*t*) are used as the measured variables, then the new $\mathbf{s} '(t)$ is supposed to be $\mathbf{s} '(t)=[x'(t+\Delta t), y'(t+\Delta t), z(t+\Delta t)]$ after “calibration”. (Here, $x'(t+\Delta t), y'(t+\Delta t)$ represent the actual value at time $t+\Delta t$.) Then we use $\mathbf{u} (t+\Delta t)=\mathbf{s} '(t)$ as the input to reconstruct $\mathbf{z} (t+2\Delta t)$. Next, we use $\mathbf{s} '(t+\Delta t)$ to “calibrate” the input and reconstruct. Finally, we repeat the above processes iteratively. Our method can greatly reduce the exponential divergence of the state variables and improve the reconstruction accuracy. In the calculation process, the number of reservoir network nodes *N* is 95, which is much lower than the reservoir network with $N=400$^18^ and $N=5000$^27^. Below, we use the Lorenz system as a example to test the “calibrated” reservoir computing.

To evaluate the reconstruction capability with the reservoir for each mode, the reconstruction accuracy of each task in the reconstruction phase is calculated with the root mean square error^34^ (RMSE)

4 $$\begin{aligned} RMSE=\sqrt{\frac{\sum (x_{re}-x_{ture})^2}{m}}, \end{aligned}$$

where $x_{re}$ is the reconstructed value of the system variable, $x_{ture}$ is the actual value of the system variable, and *m* is the total number of the reconstructed value $x_{re}$.

## Results

### Model reliability

We now investigate the reliability of the “calibrated” reservoir computing to reconstruct a chaotic system from a time series without a dynamical model. For this purpose, the simulated data from the Lorenz equation^35^ with (*x*, *y*, *z*) variables are used here.

5 $$\begin{aligned} {\left\{ \begin{array}{ll} &{}\dot{x}=A(y-x)\\ &{}\dot{y}=x(B-z)-y\\ &{}\dot{z}=xy-Cz \end{array}\right.}, \end{aligned}$$

where *A* = 10.0, *B* = 28.0, *C* = 8/3. We use the fourth-order Runge–Kutta to calculate Eq. (5). The iteration step is set as 0.02, and the maximum Lyapunov exponent^36^ is approximately 0.89 (57 steps correspond to a Lyapunov time). Setting the initial values as (1.01, 1.01, 0.0), the total iteration step is $10^{5}$, and then a $3 \times 10^{5}$ data set is obtained.

We use two different modes to perform our experiments. In case 1 (marked as the $xy-z$ mode), we choose two variables (*x*, *y*) as the measured variables to “calibrate” the input, so the other system variable (*z*) is the reconstructed variable. In case 2 ($x-yz$ mode), we choose one system variable (*x*) as the measured variable, and two system variables (*y*, *z*) as the reconstructed variables. All variables of the system are known during the entire training phase $(0< t <T_{train})$. The results demonstrate that the trained “calibrated” reservoir computing can accurately reconstruct the evolution of the reconstructed variables using any two measured state variables.

**Figure 2:**
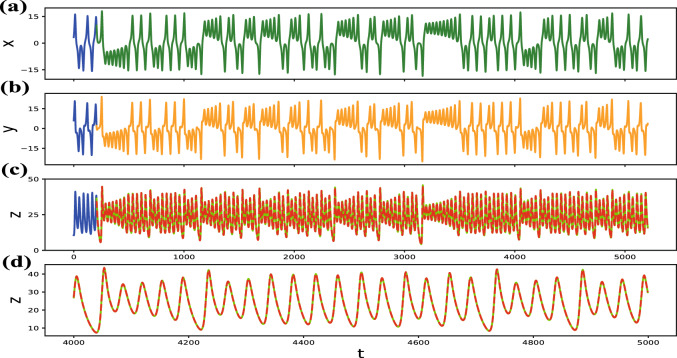
Reconstruction of the Lorenz system (Eq. 4). (**a**–**c**) The sequence diagram of variables *x*, *y*, *z* in the $xy-z$ mode, the blue solid line is the training length ($T_{train}$ = 205). For the reconstruction phase, the green and orange solid lines represent the trajectories of measured variables *x* and *y*, respectively, and the bright green solid line represents the actual value of variable *z*. The red dotted line represents the trajectories of reconstructed variable *z* calculated by reservoir computing, for $T_{test}$ = 5000. Apparently, using very little data to train the “calibrated” model, then the reconstructed variable can be reconstructed for a fairly long term. (**d**) A partial enlargement of (**c**) for t between 4000 and 5000, the dotted lines (reconstructed values) almost coincide with the solid lines (actual values), demonstrating the model’s ability to accurately reconstruct.

In Fig. 2, the *x* and *y* variables of the system are continuously recorded, as shown in (a) and (b), respectively, and the *z* variable is unmeasured which is reconstructed in (c) and (d) by the red dotted line. During the training phase, the parameters of the “calibrated” reservoir computing are as follows

**Table:** 

Number of reservoir nodes:	N = 95,
Average degree:	D = 9.5,
Bias constant:	$\xi$ = 0.1,
Spectral radius:	$\rho$ = 9.7,
Reconstruction length:	$T_{test}=10^4$.

**Figure 3:**
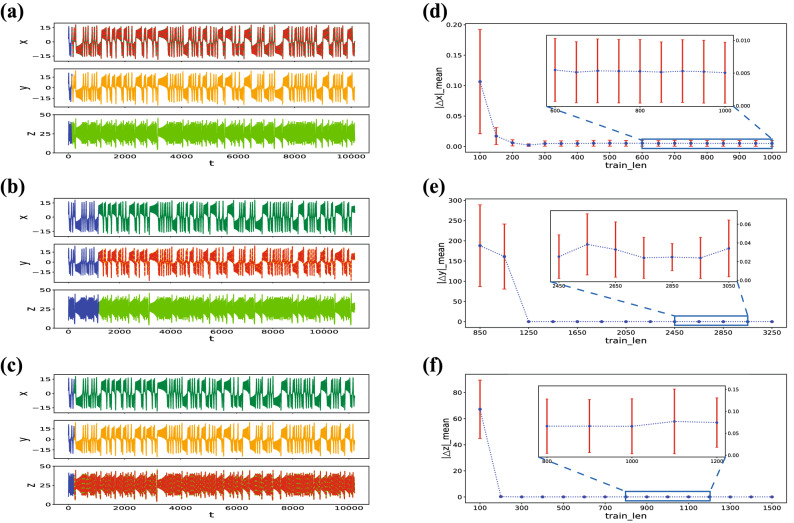
(**a**–**c**) The sequence diagram in the $yz-x$ mode, $xz-y$ mode and $xy-z$ mode, the blue solid line is the training length ($T_{train}$). The red dotted line represents the trajectories of reconstructed variables which are calculated by the “calibrated” reservoir, for $T_{test} =10^4$. (**d**–**f**) Reconstruction errors of the Lorenz system variables *x*, *y*, and *z* in the three modes: $yz-x$ mode, $xz-y$ mode and $xy-z$ mode, respectively. The blue dots represent the corresponding average values of the absolute difference between the reconstructed and actual value. The red error bars are the standard deviation values of $|\Delta x |$, $|\Delta y |$, $|\Delta z |$. The subgraphs are partially enlarged views of (**d**–**f**). All modes’ reconstruction error falls dramatically at initially, then it reaches a stable value as training length increases. (Without special instructions, the blue dots and red error bars remain the same definition.).

The root mean square error (RMSE) of the system reconstructed variable *z* is 9.998e−2, indicating a very small deviation between the reconstructed values and the actual values. Similarly, we calculated the shortest training length ($T_{test} =10^4$, RMSE $< 0.1$) of the Lorenz system when the reconstructed variable is *x* or *y*, and the results are shown in Fig. 3 and Table 1. It should be noted, the shortest training length is used as an index of the the efficiency of reconstruction when the reconstruction length is fixed and RMSE $< 0.1$. The results indicate that the proposed “calibrated” reservoir computing method can give a reliable reconstruction as the variables have a long reconstruction length and small RMSE.

**Table 1:** Shortest training length of the Lorenz system in the case 1($T_{test} =10^4$, RMSE $< 0.1$).

Mode style	$Min(T_{train})$	$T_{test}$
Lorenz: $yz-x$	125	10,000
Lorenz: $xz-y$	1205	10,000
Lorenz: $xy-z$	205	10,000

### Relationship between reconstruction and training length

In this section, we study the relationship between the error of the reconstructed variables and the training length. We give different lengths of the training data and observe the changes in reconstruction errors under the modes of $yz-x$, $xz-y$, and $xy-z$. The results are shown on the right side of Fig. 3. The blue dots represent the average values of the absolute differences between the reconstructed values and the actual values ($|\Delta x |=abs(x_{re}-x_{ture})$) at different training lengths with $T_{test} = 10^4$. The red error bars indicate the standard deviation values of $|\Delta x |$, $|\Delta y |$, and $|\Delta z |$. As shown in Fig. 3, the reconstruction error drops sharply when the training length reaches a certain value. After that, the reconstruction error is maintained. This means that the “calibrated” reservoir computing has a limit of accuracy, and it is not necessary to pursue a longer training length.

**Figure 4:**
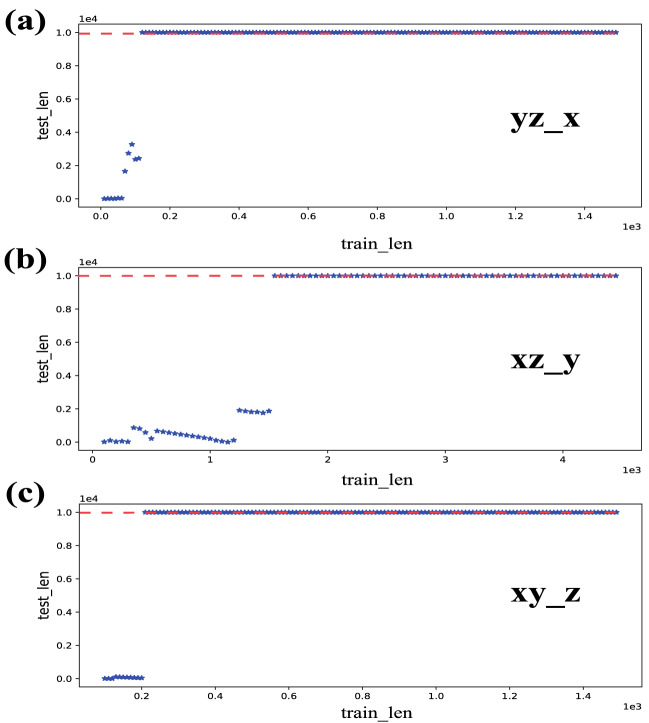
(**a**–**c**) Tendency of the longest $T_{test}$ of variable *x*, *y*, *z* versus $T_{train}$ in each mode, when the RMSE of the Lorenz system $< 0.1$. The Max($T_{test}$) has been set at $10^{4}$. The blue star dots are the longest reconstructable length. The further proof of the relationship between $T_{train}$ and $T_{test}$ is proved shows that these three modes all have a threshold.

Then, the relationship between training length and reconstructable length is studied. If $T_{test}$ reaches $10^{4}$, then RMSE is still less than 0.1, the reconstruction length is cut off as it is equivalent to approximately 200 Lyapunov time in the Lorenz system, and the “reconstructable length” is set as $10^{4}$. However, if RMSE is over 0.1 before $T_{test}$ reaches $10^{4}$, then the “reconstructable length” is set as the longest reconstruction length when RMSE $< 0.1$. In Fig. 4, we can see that the reconstructable lengths of the three modes ($yz-x$, $xz-y$ and $xy-z$) are basically below 5000 steps before the training length reaches a certain value. Once the threshold is reached, the system generates a jump mutation, and $T_{test}$ ascends to the maximum value $10^{4}$. These results are identical to those obtained from the study of the reconstruction error and training length. These models’ various thresholds confirm that the quantity of information carried by different system variables is diverse.

**Figure 5:**
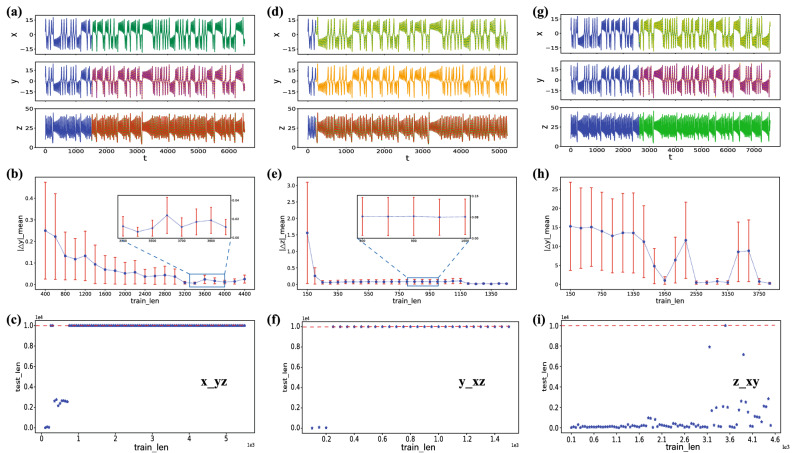
Reconstruction results of the Lorenz system in the corresponding $x-yz$ mode, $y-xz$ mode and $z-xy$ mode. (**a**–**c**) The sequence diagram, the reconstruction error, and the longest reconstructable length about the $x-yz$ mode, respectively. (**d**–**f**) The same results obtained in the $y-xz$ mode. Similarly, (**e**–**i**) The outcomes under the $z-xy$ mode. In (**a**,**d**,**g**), the dotted lines are the reconstruction results of the “calibrated” reservoir computing, the real lines are the actual values of system variables (the blue real lines represent the training length). All results represent the “calibrate” model can work well with different modes. (**b**,**e**,**h**) The reconstruction error of three modes in the Lorenz system, the front two modes have a sharply declining trend with increasing $T_{train}$, the reconstruction error of the last mode decreases after reaching a certain training length, but there is some fluctuation. (**c**,**f**,**i**) The tendency of longest $T_{test}$ versus $T_{train}$ when RMSE $< 0.1.$ In (**i**), the longest $T_{test}$ does not have a certain threshold, the variables *x*, *y* can only be reconstructed accurately by some $T_{train}$ values. The basic reservoir parameters we use here are the same for Figs. 2, 3 and 4.

We consider reducing the measured variables based on the preceding experimental results: the number of measured variables is decreased to one. After constructing $x-yz$, $y-xz$, and $z-xy$ modes, the same method as above is performed. The results are shown in Fig. 5 and Table 2.

**Table 2:** Shortest training length of the Lorenz system in the case 2($T_{test} =10^4$, RMSE $< 0.1$).

Model style	$Min(T_{train})$	$T_{test}$
Lorenz: $x-yz$	1520	10,000
Lorenz: $y-xz$	208	10,000
Lorenz: $z-xy$	2618	10,000

The results show that “calibrated” reservoir computing can still reconstruct well when the measured variables are reduced. Meanwhile, the shortest training length is the fewest in the $y-xz$ mode when the reconstruction length is the same. However, Fig. 5i shows that there is no certain threshold in the $z-xy$ mode for the reservoir to make a stable reconstruction. It implies that variable *z* may contain less information than other variables. Overall, although the shortest training length for the reconstructable length varies in six modes, its training length is much shorter than the reconstructable length.

### Relationship between reconstruction and underlying dynamical features

In this section, we study the the efficiency of reconstruction under different data sets taken from many classical chaotic systems, and compare the results in detail. For comparison, we also select the shortest training length as the index of reconstruction for each system. The fewer the shortest training length we need, the better the reconstruction.

In case 1($xy-z$), we found that the variables that coupled most with others (nonlinear terms) in dynamical terms can be best reconstructed, as shown in Table 1 of the Lorenz model. In Lorenz system Eq. (5), the nonlinear terms are *xy* and *xz*, *x* is coupled with the other two variables. Therefore, the $yz-x$ mode has the best reconstruction, as *x* is set as the reconstructed variable. This rule is robust, and we demonstrated for other models as shown in the Supplementary Materials.

In case 2($x-yz$), we found that the more frequently variables appear in other equations, the better reconstruction when it is used as the measured variable to reconstruct other variables. Take the Rössler system^37^ as an example:

6 $$\begin{aligned} {\left\{ \begin{array}{ll} &{}\dot{x}=-y-z\\ &{}\dot{y}=x+0.15y\\ &{}\dot{z}=0.2+z(x-10). \end{array}\right. } \end{aligned}$$

Here, variable *x* takes part in the dynamical revolutions of both $\dot{y}$ and $\dot{z}$, so variable *x* has the best reconstruction results when it is used as the measured variable. The results is shown in Table 3. To further support the rule, we tested many systems and verified that these systems also follow this rule. These results are shown in the Supplementary Materials.

**Table 3:** Shortest training length of the Rössler system in the case 1 ($T_{test} = 10^4$, RMSE $< 0.1$).

Model style	$Min(T_{train})$	$T_{test}$
Rössler: $x-yz$	1671	10,000
Rössler: $y-xz$	1705	10,000
Rössler: $z-xy$	2225	10,000

In addition, the reconstruction can reveal dynamical structures when the data are measurable while the equation is unknown. For example, if the $x-yz$ mode has the best reconstruction, we can conversely speculate that there may be *x* related terms in $\dot{y}$ and $\dot{z}$. If the $xy-z$ mode has the best reconstruction, then *z* may have nonlinear terms in the evolutive equations of *x* and *y*.

### Feasibility of “calibrated” reservoir computing under interval “calibration”

**Figure 6:**
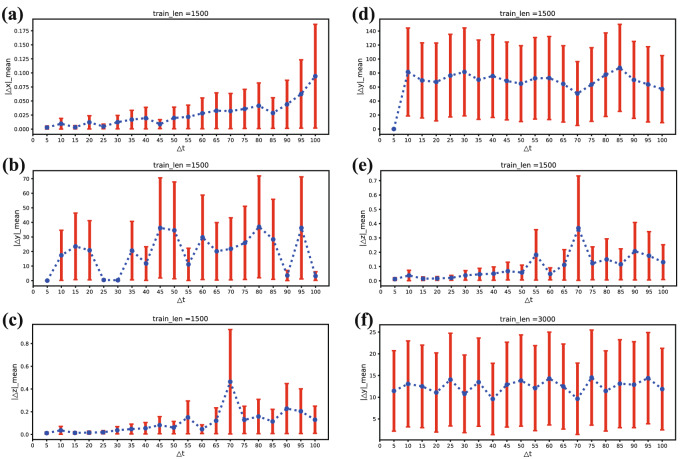
The reconstruction error of the Lorenz system versus the interval $\Delta t$ (5–100) in the six modes. (**a**–**e**) The tendency of the reconstruction error and the interval $\Delta t$ in the five modes (i.e., $yz-x$ mode, $xz-y$ mode, $xy-z$ mode, $x-yz$ mode, and $y-xz$ mode) when $T_{train}$ = 1500. All the five modes can reconstruct well when $\Delta t$ = 5, and the reconstruction of $yz-x$ mode is the best. Unfortunately, the reconstruction of some modes (i.e., $xz-y$, $x-yz$) become worse when $\Delta t$ >5. In (**f**), the reconstruction error (RMSE) for the $z-xy$ mode is always much greater than 0.1 even when $T_{train}$ = 3000. It signifies that $z-xy$ mode cannot work well under interval calibrating.

In this section, we consider that the variables may be spare sampling instead of continuously as continuous measuring may cost too much or cause damage to systems. We select “calibrating” the input at intervals for the sake of information utilization. We first set the interval length as $\Delta t=5,10,\ldots ,95,100$. The distribution of $\Delta t$ and the reconstruction error corresponding to different modes under the Lorenz system are shown in Fig. 6. The reconstruction error increases slightly but the distribution of error becomes wider.

**Figure 7:**
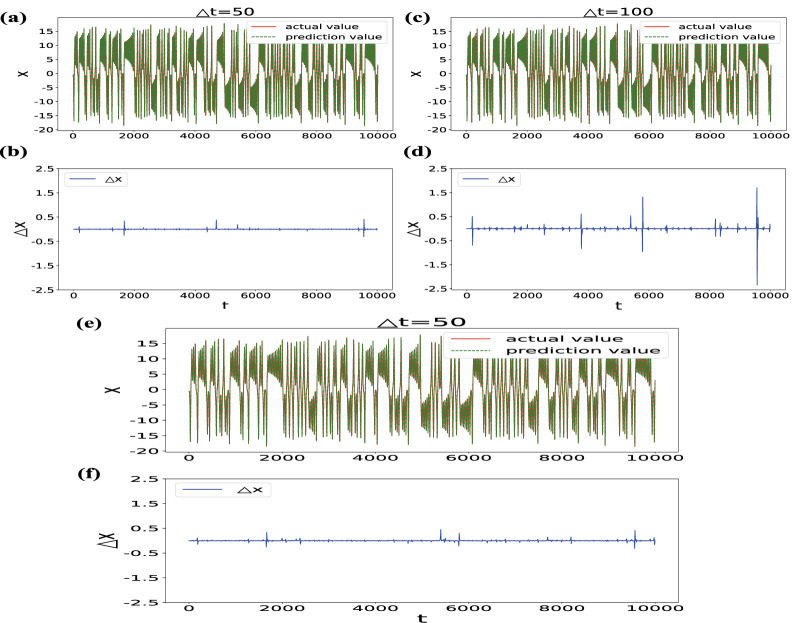
The sequence diagram shows the reconstruction results of the reconstructed variables and the difference between the reconstructed values and the actual values ($\Delta t$ = 50, 100, respectively). (**a**,**c**) The comparison between the actual variable *x* (red solid line) and the corresponding reconstruction results (green dashed line), for the $yz-x$ mode. (**b**,**d**) The sequence diagrams of $\Delta x$ under the $yz-x$ mode. The $\Delta x$ maintains less than 0.5 in (**b**), the $\Delta x$ in (**d**) is always small except in several cases. (**e**,**f**) The sequence diagram of the reconstruction results and the difference $\Delta x$, corresponding to the reconstructed variable *x* ($\Delta t$ = 50, the $y-xz$ mode). In (**f**), the difference $\Delta x$ is basically around $\Delta x=0$.

For a precise overview, the reconstruction results of the “calibrated” reservoir computing when $\Delta t=50$ and $\Delta t=100$ are shown in Fig. 7a–d. When the interval step is 50 or 100, the reconstructed values are nearly coincident with the actual value, and RMSE are both less than 0.1. However, the reconstruction error at $\Delta t=100$ is obviously larger than $\Delta t=50$, and the maximum error $\Delta x$ reaches 2.35 (within $T_{test}=10^4$). It means that the “calibrated” reservoir computing with interval sampling works well but the reconstruction accuracy trends decrease when $\Delta t$ is too large.

Figure 7e shows the time sequence diagram of the reconstructed value by the “calibrated” reservoir computing (green dashed line) and the actual value by numerical calculation (red solid line) in the $y-xz$ mode. These results demonstrate that the two datasets are essentially in agreement and the RMSE value is 2.26e−2. In Fig. 7f, the difference $\Delta x$ is approximately $\Delta x=0$, the “calibrated” reservoir reservoir computing can still accurately work in the $y-xz$ mode. It implies that the calibrating variables can be simplified to intervals instead of continuous while the reconstruction can be maintained at most times.

## Conclusion

We proposed “calibrated” reservoir computing by calibrating the input to reconstruct the evolution of reconstructed variables under advanced conditions (“reservoir observers”^18^). Then, we discussed the efficiency of reconstruction in the absence of a mathematical model for dynamic systems when the variables are partially measured. Our results can be summarized as follows.

1. The reconstruction length can be extended to $10^4$ or even longer in our “calibrated” method, as shown in Fig. 2. Compared to 5-6 Lyapunov times of the classical model^25^ and 12 Lyapunov times of the hybrid model^26^, it is significantly promoted, and the training length can be obviously reduced. The more measured variables we use, the better reconstruction results (Tables 1, 2). Moreover, the results are robust in different chaotic dynamical systems such as the high dimension coupled system, the Rössler system, the Lorenz system, and so on as shown in the Supplementary Materials.
2. There is a threshold between the training length and the reconstructable length in most modes. The reconstruction has a limit in accuracy, as its error (or reconstructable length) is maintained when the training length increases to a certain value.
3. The activities in other’s dynamical terms and the types of interaction affect the reconstruction. The more it interacts with others in dynamical equations, the better reconstruction it makes when it is chosen as the measured variable. The nonlinear terms can reveal more information than linear terms once the interacting terms are equal.
4. When the measured variables are sparse, our methods are still valid (see Fig. 7).

Overall, when all system variables can be recorded for a short period and partial variables can be continually or discretely measured for a long period, the “calibrated” reservoir computing we proposed and investigated here can effectively reconstruct the remaining reconstructed variables by using the measured data. The reconstruction is maintained in different chaotic systems. Moreover, if all the data are measured, then the reconstruction varies via different input measured variables, which can reveal the underlying dynamical features.

## Supplementary Information

Supplementary Information.

## Data Availability

All data generated and analysed in the manuscript are reproducible based on the algorithms detailed in the article (see “Model” and the “Results” sections).
