# Supplementary material for: Time series reconstructing using calibrated reservoir computing
Source: Sci Rep. 2022 Sep 29;12:16318. doi: 10.1038/s41598-022-20331-3 (PMC9522934; doi:10.1038/s41598-022-20331-3)
Supplement: Supplementary file 1 — Supplementary Information. [file 41598_2022_20331_MOESM1_ESM.pdf]

Supplementary Material for "Time Series Reconstructing Using Calibrated Reservoir Computing"

Yeyuge Chen[1], Yu Qian[2], and Xiaohua Cui[1,*]

[1]School of Systems Science, Beijing Normal University, Beijing 100875, China
[2]Nonlinear Research Institute, Baoji University of Arts and Sciences, Baoji 721007, China
[*]Correspondence should be addressed to (xhcui@bnu.edu.cn)

1 Reconstruction results of the coupled Lorenz system in different states

1.1 Reconstruction results of the 6-dimensional system

We extend the application system to the 6-dimensional coupled Lorenz system, which is composed of a one-dimensional lattice with two chaotic Lorenz systems. The specific calculation equations are as follows:

$$\begin{cases} \dot{x}1 = 10(y1-x1)-\sigma(x2-x1) \\ \dot{y}1 = x1(28-z1)-y1 \\ \dot{z}1 = x1y1-8/3z1 \\ \dot{x}2 = 10(y2-x2)-\sigma(x1-x2) \\ \dot{y}2 = x2(28-z2)-y2 \\ \dot{z}2 = x2y2-8/3z2 \end{cases}, \tag{S1}$$

where $\sigma = 0.01$. Setting the initial values as (10.03, 14.69, 22.11, 10.04, 14.68, 22.10), the iteration step is set as 0.002, the total iteration step is 10^5, and then a 6×10^5 data set is obtained.

Using the 6-dimensional data obtained from the numerical calculation of the system, we also reconstructed the data by using the "calibrated" reservoir computing. In the 6-dimensional coupled Lorenz system, we use $x1$ and $x2$ or $y1$ and $y2$ or $z1$ and $z2$ in the system as one variable to reconstruct another variable. For example, in the model $yz-x$, $y1,y2,z1,z2$ are used as the measured variables, and the reconstructed values are $x1,x2$. The results of different modes in the coupled Lorenz system are shown in Fig. S1 and Table SI. We demonstrate that using our proposed "calibrated" reservoir computing can make an accurate reconstruction of the coupled Lorenz system in different states. However, it is obvious that the reconstruction error of $x-yz$ mode is much larger than that in other modes. It may be caused by the coupling of variable x, and the training set is larger when variables are coupled than without coupling, such as $xy-z$ and $xz-y$ modes. With the increase of spatial coupling part and dimension, maintaining the same reconstruction length, a larger training set needs to be added, otherwise the reconstruction length will become shorter.

Table SI. Shortest training lengths of the coupled Lorenz system($T_{test}=10^3$, $RMSE$ <0.1)

Model style	$Min(T_{train})$	T_{test}	Model style	$Min(T_{train})$	T_{test}
$xy-z$	2600	1000	$x-yz$	2500	1000
$xz-y$	2500	1000	$y-xz$	2600	1000
$yz-x$	1600	1000	$z-xy$	2900	1000

Furthermore, we consider the case where two subsystem variables are used independently, *i.e.*, $x1y1z1$ (or $x2y2z2$) of one system is used to reconstruct $x2y2z2$ (or $x1y1z1$) of the other. The results of different modes in the coupled Lorenz system are shown in Fig. S2 and Table SII. Its findings demonstrate that regardless of which side of the variable is used to reconstruct the other side, the reconstruction effect is essentially the same. This result could be explained by the fact that the parameter settings of the two subsystems are consistent, so there is no difference in the reconstruction effect. It should be noted that RMSE in all tables is the average of all reconstructed variables.

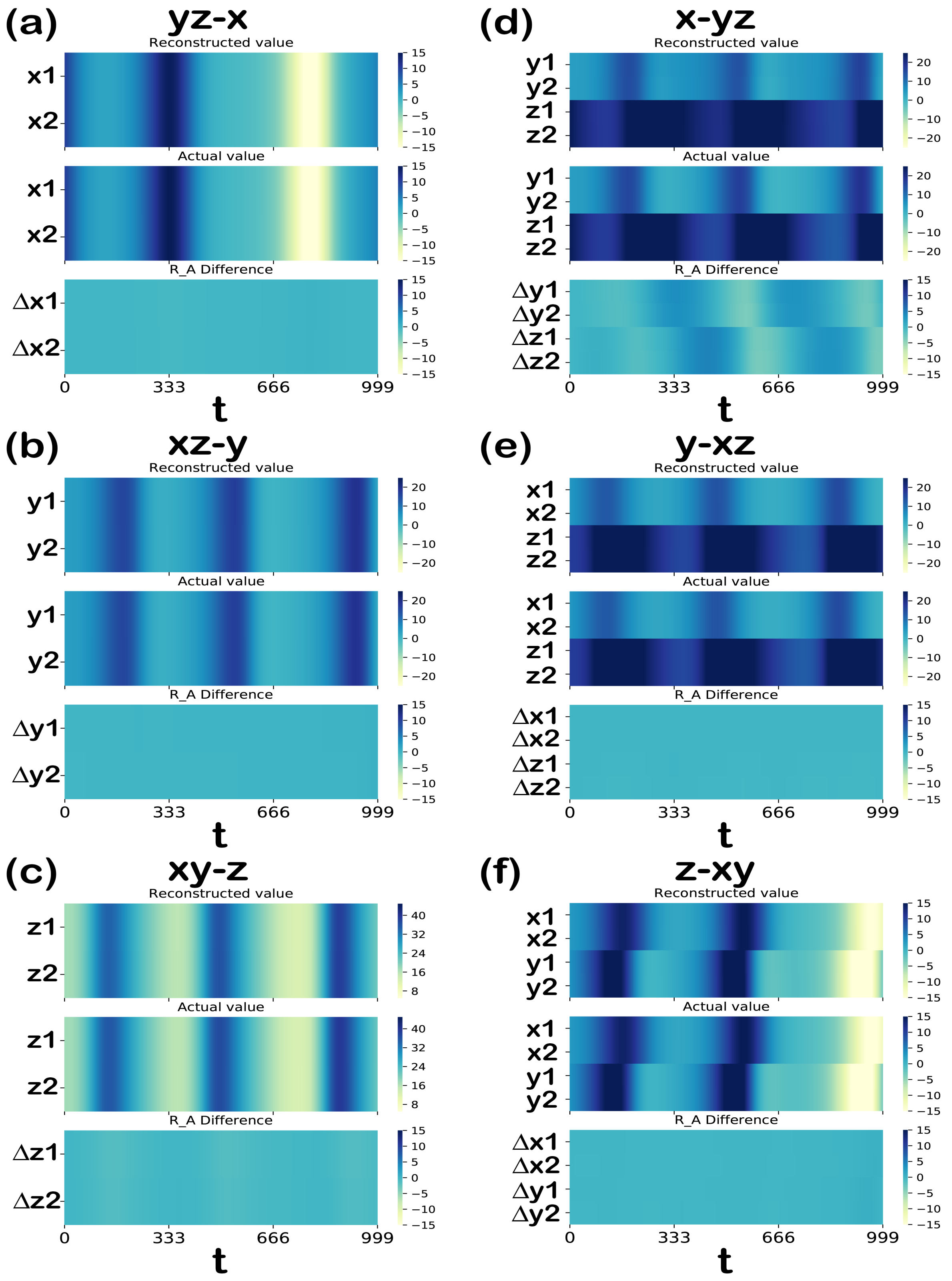

Figure S1. The reconstruction results of the coupled Lorenz system with $\sigma = 0.01$, using a single reservoir of size $D_R = 100$. (a)-(f) are the reconstructed values, actual values and the difference between them in the six modes. The first graph of all subgraphs is the reconstruction result, the second is the actual value, and the third is the difference between them.

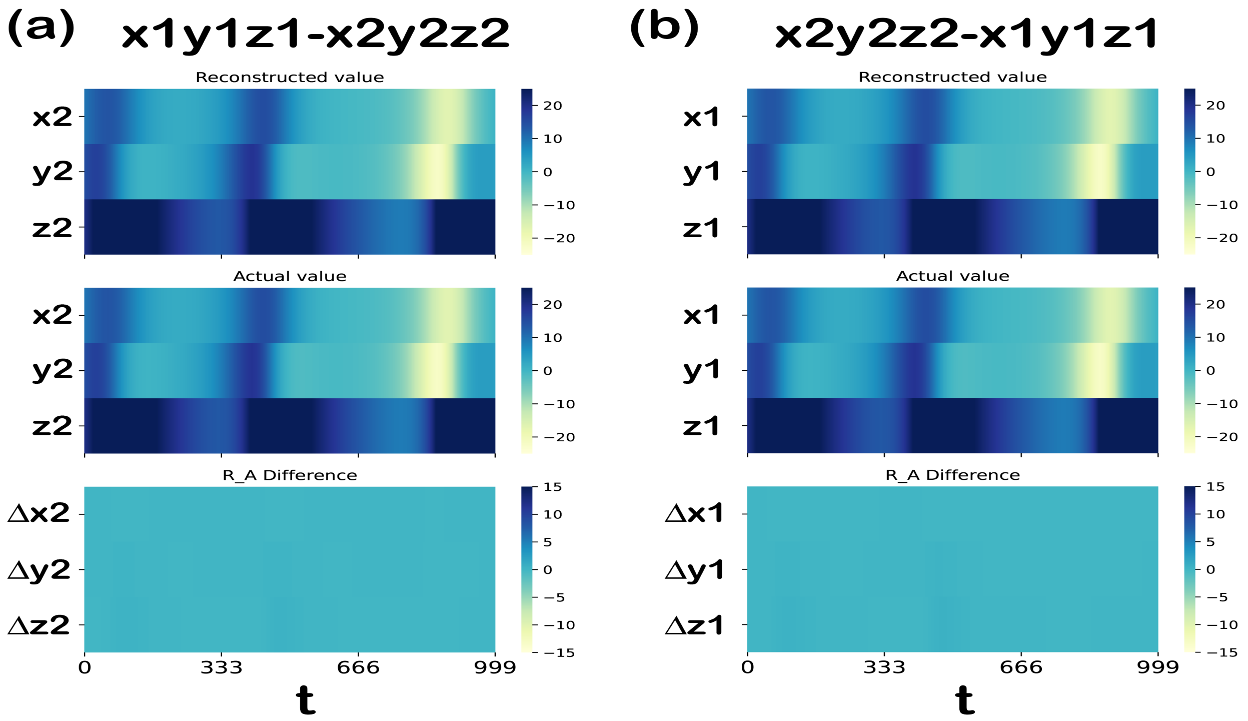

Figure S2. The reconstruction results of the 6-dimensional coupled Lorenz system in the $x1y1z1-x2y2z2$ mode and $x2y2z2-x1y1z1$ mode. (a) and (b) are the reconstructed values, actual values, and the difference between them in the two modes. The first graph of all subgraphs is the reconstruction result, the second is the true value, and the third is the difference between them.

Table SII. Shortest training lengths of the coupled Lorenz system($T_{test}=10^3$, $RMSE$ <0.1)

Model style	$Min(T_{train})$	T_{test}	$RMSE$
$x1y1z1-x2y2z2$	1500	1000	0.0696
$x2y2z2-x1y1z1$	1500	1000	0.0744

1.2 Reconstruction results of the 15-dimensional system

In this section, we extend the "calibrated" reservoir computing in the 15-dimensional Lorenz system to study the relationship between the performance of reconstruction and the number of the measured variables. Specific parameter settings are as follows:

$$\begin{cases} \dot{xi}=10(yi-xi)-\sigma/2(xj-xi) \\ \dot{yi}=xi(28-zi)-yi \\ \dot{zi}=xiyi-8/3zi \end{cases}, \tag{S2}$$

where $j \neq i$, $i,j=1,2,3,4,5$, $\sigma=0.01$. Using the obtained data, we have conducted a detailed study on the reconstruction performance of the "calibrated" reservoir on high-dimensional systems. The results are shown in Fig. S3 and Table SIII. As the dimension of the system grows, so does the calculation difficulty. We choose the $yz-x$ mode with the best reconstruction effect according to the calculation results, x,y,z respectively represent all xi,yi,zi in each subsystem.

Then, we try to reduce the number of the measured variables to make an accurate reconstruction in the $yz-x$ mode, *i.e.*, reduce the number of the measured variables yz. In the $yz-x-1$ mode, for example, the measured variables are $y1,z1$, and the reconstructed variables are $x1,x2,x3,x4,x5,y2,y3,y4,y5$ and $z2,z3,z4,z5$. In the $yz-x-2$ mode, the measured variables are $y1,y2,z1,z2$, and the reconstructed variables are $x1,x2,x3,x4,x5,y3,y4,y5$ and $z3,z4,z5$. The modes $yz-x-3$ and $yz-x-4$ are also similar. The specific outcomes are as follows: The system's reconstruction effect appears to weaken as the number of the measured variables decreases. However, whether in a single or coupled system, we find that if the yz variable of the subsystem

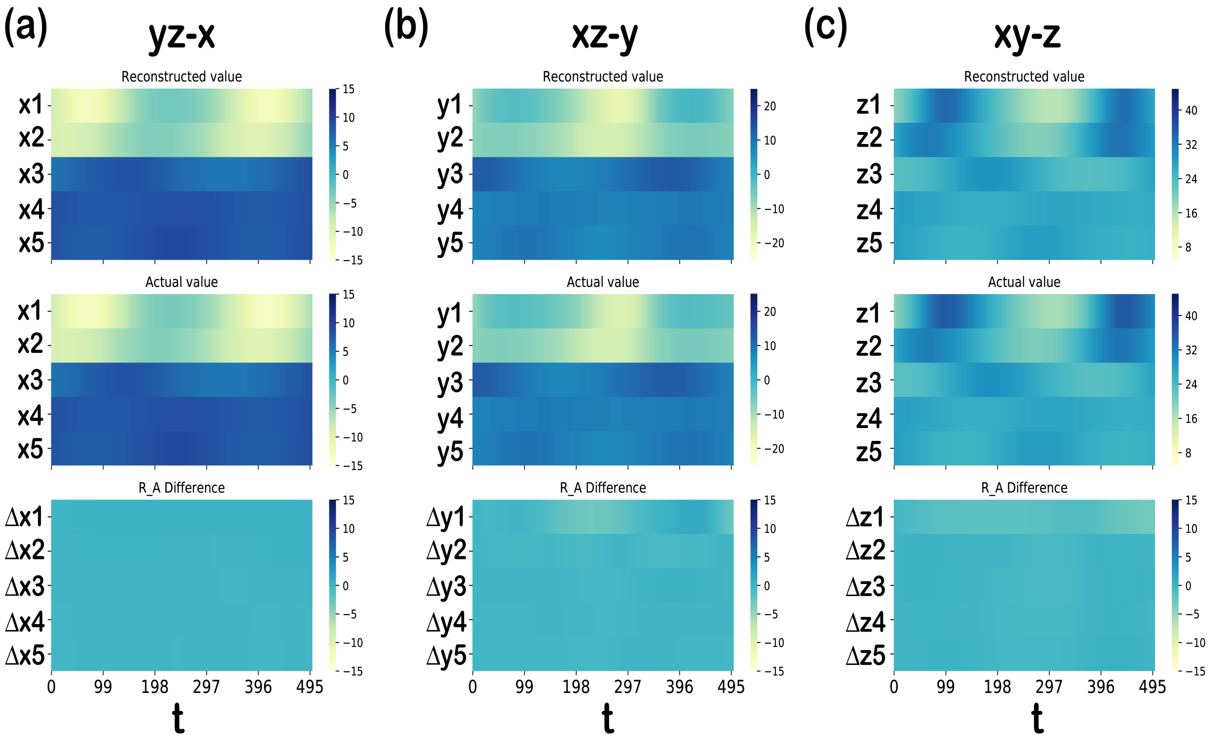

Figure S3. The reconstruction results of the 15-dimensional coupled Lorenz system in the $yz-x$ mode, $xz-y$ mode and $xy-z$ mode. (a)-(c) are the reconstructed values, actual values, and the difference between them in the three modes.

Table SIII. Shortest training length of the 15-dimensional coupled Lorenz system($T_{test}=500$)

Model style	$Min(T_{train})$	T_{test}	$RMSE$
$yz-x$	1400	500	0.049
$xz-y$	1500	500	0.408
$zy-z$	1400	500	0.538

is given, the x variable of the subsystem can be reconstructed. This also implies that "calibrated" reservoir computing may be better able to obtain the interaction of various variables within the system, whereas learning the spatial coupling relationship may be more difficult. Finally, after testing each model, we can reach the following conclusions: When the reconstruction length T_{test} is 500, the number of observed variables in the 15-dimensional coupling system must be approximately 53.3%(8/15) in order to achieve accurate reconstruction. The results are shown in Fig. S4 and Table SIV. When T_{test} is 100, the number of the measured variables required to achieve accurate reconstruction is only about 26.7%(4/15). The results are shown in Fig. S5 and Table SV.

In addition, we also constructed the xyz mode, that is, using the xyz variables of several of the five subsystems to reconstruct the xyz variables of the remaining subsystems. For example, in the $xyz-1$ mode, the measured variables are $x1, y1$ and $z1$, while the remaining variables $x2, x3, x4, x5, y2, y3, y4, y5$ and $z2, z3, z4, z5$ are the reconstructed variables. The measured variables in the $xyz-2$ mode are $x1, x2, y1, y2$ and $z1, z2$, while the remaining variables are $x3, x4, x5, y3, y4, y5$ and $z3, z4, z5$. The $xyz-3$ and $xyz-4$ modes are interchangeable. When the reconstruction length T_{test} is set as 500, the number of the measured variables needs to reach 80%(12/15) to accurately reconstruct the 15-dimensional system. When the reconstruction length T_{test} is set to 100, at least 40%(6/15) measured variables are required for accurate reconstruction. The results are shown in Fig. S6, Fig. S7 and Table SVI, Table SVII. From the above experimental results, we can see that the reconstruction error RMSE of all modes has been significantly reduced with the shortening of T_{test}. It also implies that in a higher-dimensional system, if we want to perform accurate reconstruction for a long time, we can increase T_{train} appropriately. Furthermore, the requirement for accurate reconstruction in xyz mode is far greater than in $yz-x$ mode, indicating that the x variable is unimportant in the reconstruction of this system. All the above experimental results further demonstrate that the "calibrated" reservoir computing can still perform accurate reconstruction in high-dimensional systems.

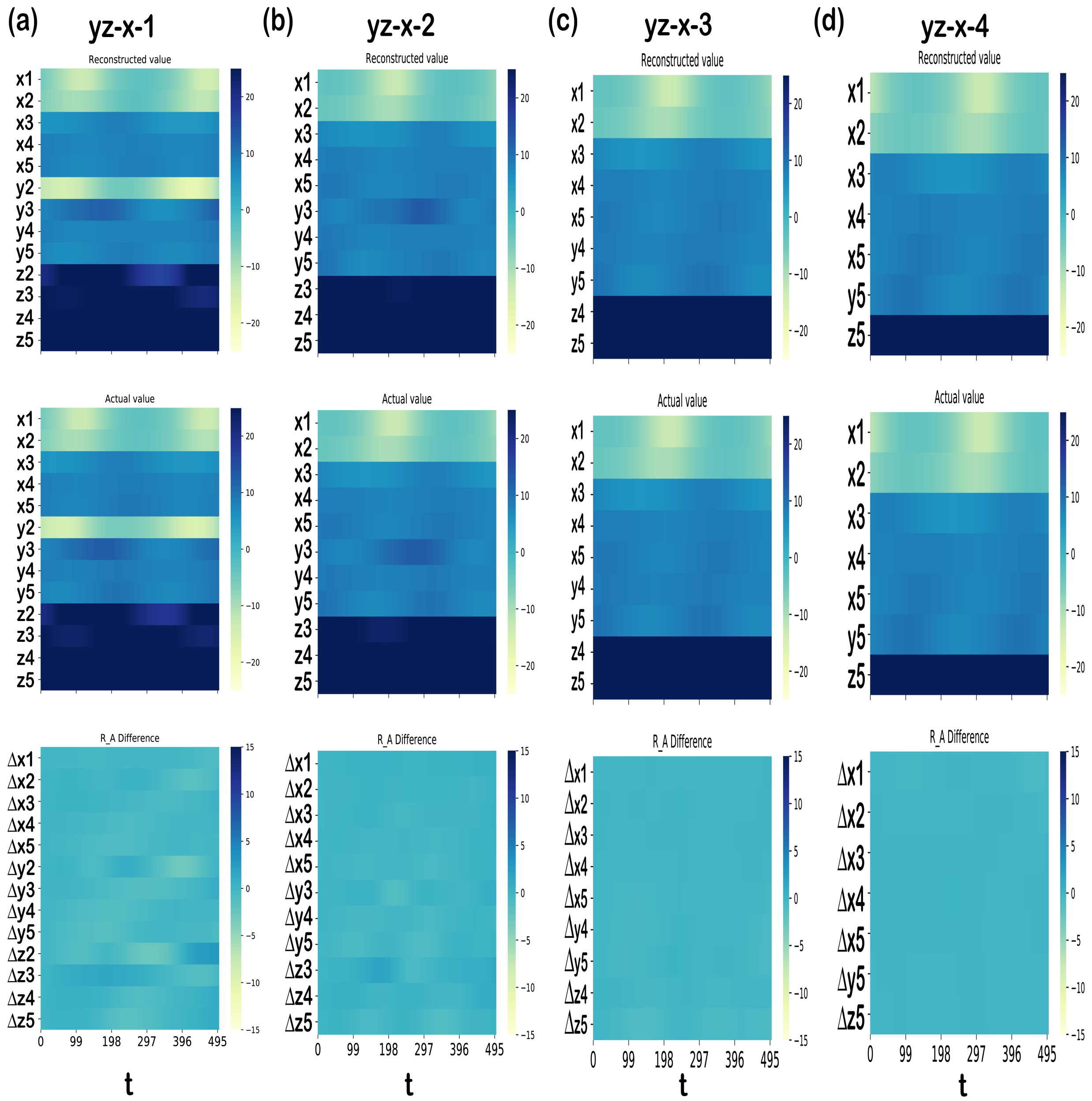

Figure S4. The reconstruction results of the 15-dimensional coupled Lorenz system in the $yz-x-1$ mode, $yz-x-2$ mode, $yz-x-3$ mode and $yz-x-4$ mode when the reconstruction length T_{test} is 500. (a)-(d) are the reconstructed values, actual values, and the difference between them in these modes.

Table SIV. Shortest training length of the 15-dimensional coupled Lorenz system($T_{test} = 500$)

Model style	$Min(T_{train})$	T_{test}	$RMSE$
$yz-x-1$	1700	500	0.774
$yz-x-2$	1600	500	0.431
$yz-x-3$	1600	500	0.242
$yz-x-4$	1500	500	0.096

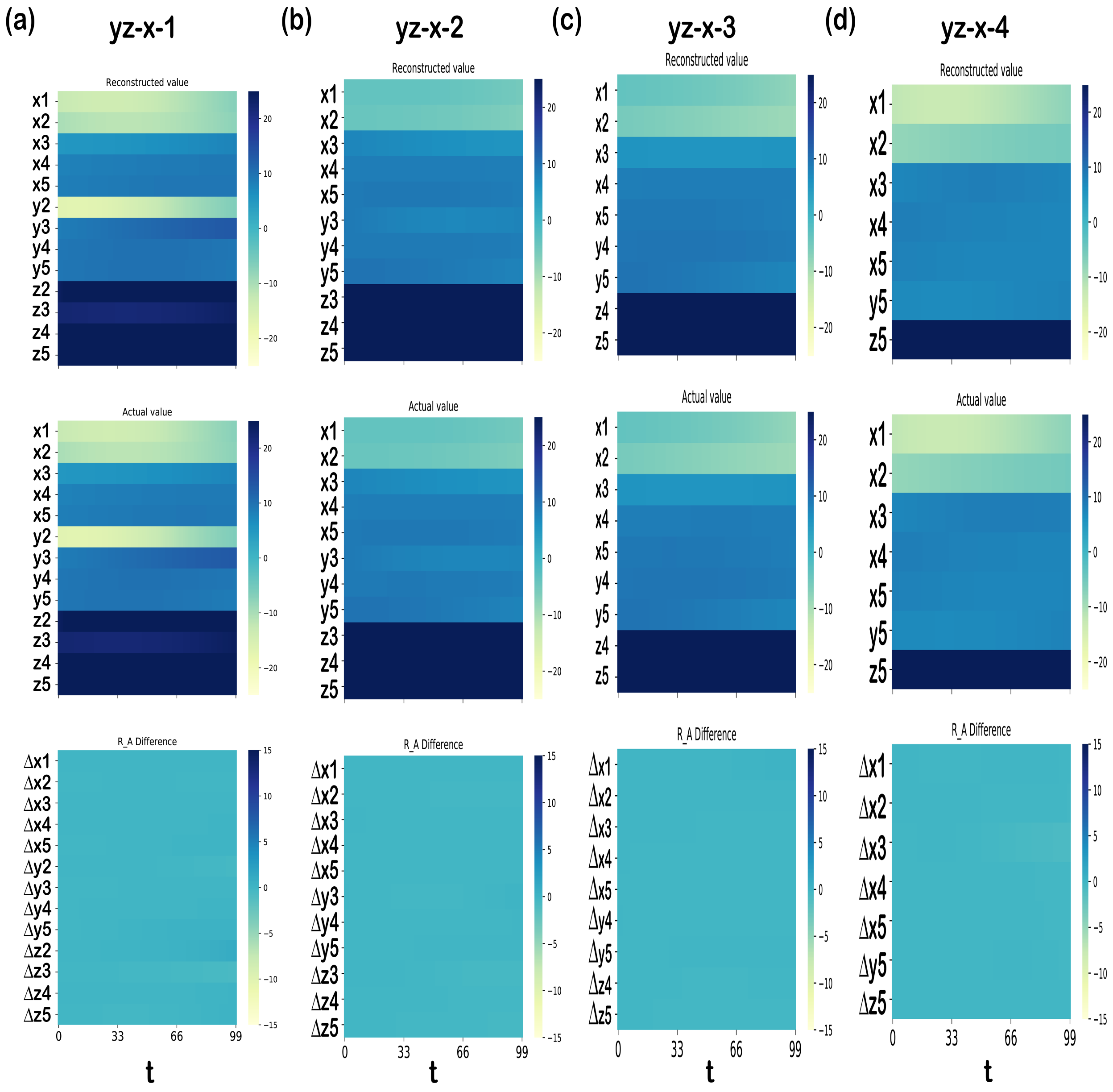

Figure S5. The reconstruction results of the 15-dimensional coupled Lorenz system in the $yz-x-1$ mode, $yz-x-2$ mode, $yz-x-3$ mode and $yz-x-4$ mode when the reconstruction length T_{test} is 100. (a)-(d) are the reconstructed values, actual values, and the difference between them in these modes.

Table SV. Shortest training length of the 15-dimensional coupled Lorenz system($T_{test} = 100$)

Model style	$Min(T_{train})$	T_{test}	$RMSE$
$yz-x-1$	2500	100	0.144
$yz-x-2$	1600	100	0.098
$yz-x-3$	1300	100	0.075
$yz-x-4$	1100	100	0.079

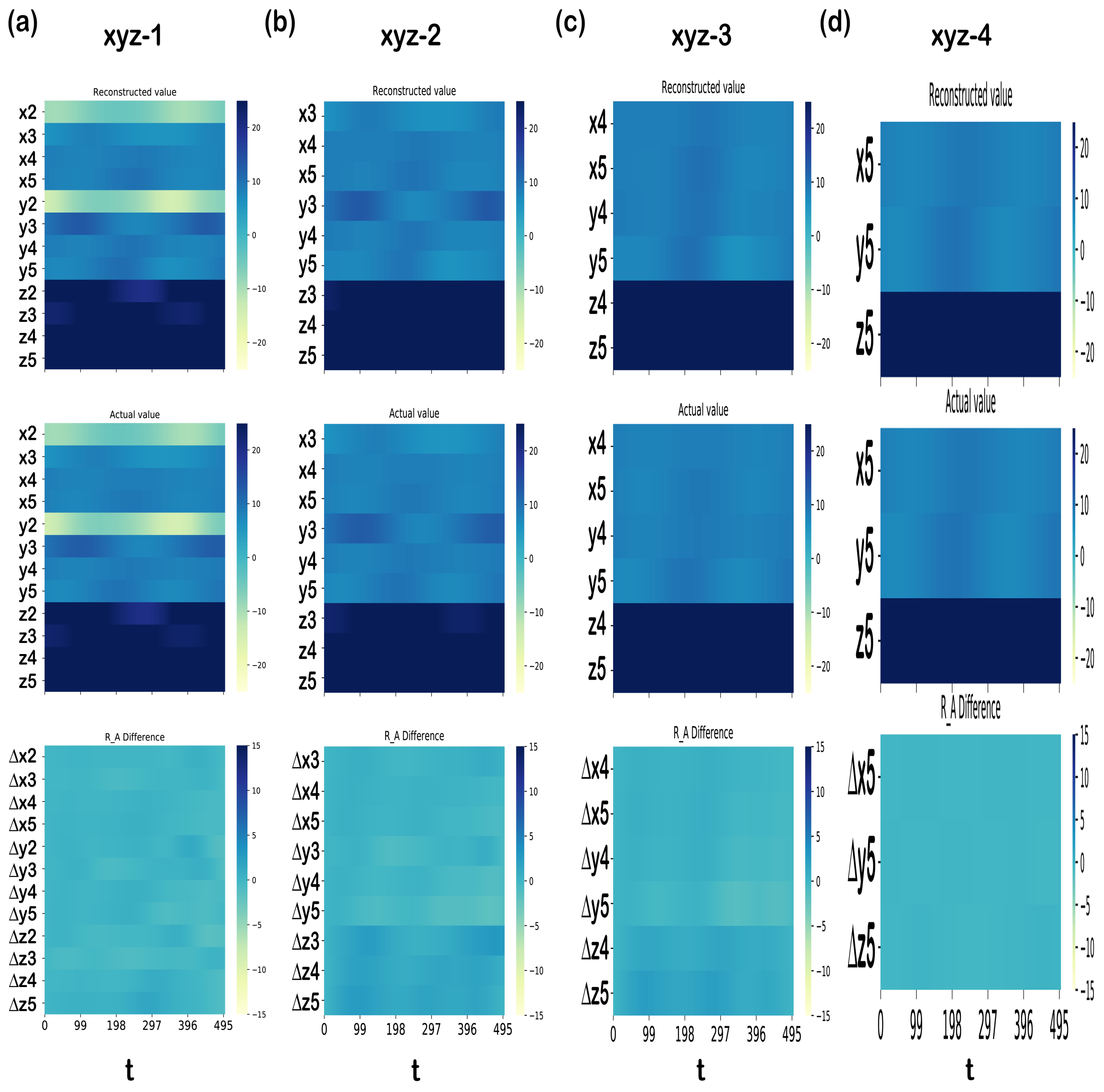

Figure S6. The reconstruction results of the 15-dimensional coupled Lorenz system in the $xyz-1$ mode, $xyz-2$ mode, $xyz-3$ mode and $xyz-4$ mode when the reconstruction length T_{test} is 500. (a)-(d) are the reconstructed values, actual values, and the difference between them in these modes.

Table SVI. Shortest training length of the 15-dimensional coupled Lorenz system($T_{test} = 500$)

Model style	$Min(T_{train})$	T_{test}	$RMSE$
$xyz-1$	1400	500	0.453
$xyz-2$	1400	500	0.221
$xyz-3$	1400	500	0.204
$xyz-4$	1400	500	0.083

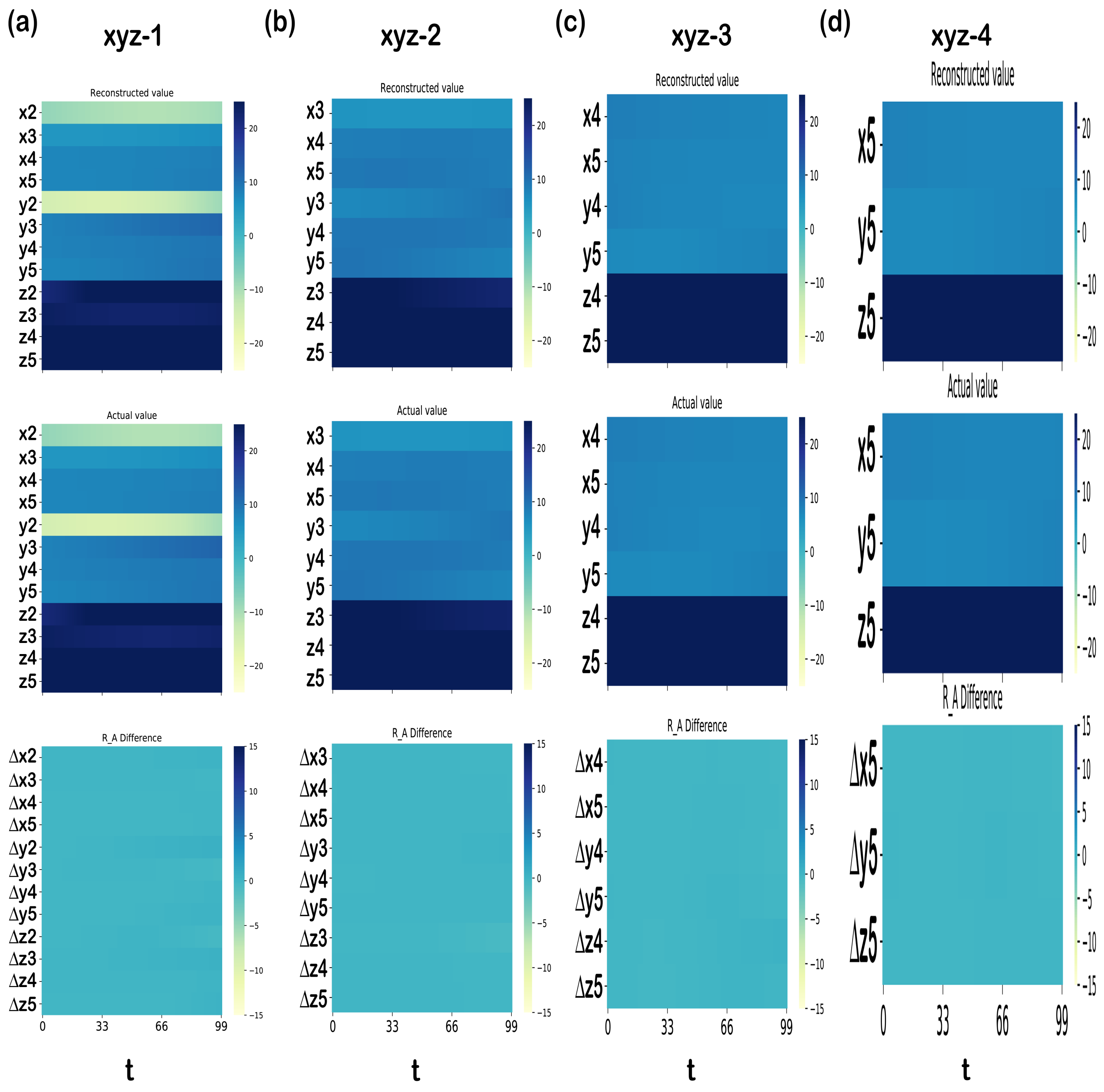

Figure S7. The reconstruction results of the 15-dimensional coupled Lorenz system in the $xyz-1$ mode, $xyz-2$ mode, $xyz-3$ mode and $xyz-4$ mode when the reconstruction length T_{test} is 100. (a)-(d) are the reconstructed values, actual values, and the difference between them in these modes.

Table SVII. Shortest training length of the 15-dimensional coupled Lorenz system($T_{test} = 100$)

Model style	$Min(T_{train})$	T_{test}	$RMSE$
$xyz-1$	2100	100	0.121
$xyz-2$	1300	100	0.055
$xyz-3$	1100	100	0.095
$xyz-4$	1100	100	0.062

2 Reconstruction results of Rössler systems in different states

We demonstrate that using our proposed "calibrated" reservoir computing can make an accurate reconstruction of the classical Rössler[1] system in different states.

$$\begin{cases} \dot{x} = -y - z \\ \dot{y} = x + Ay \\ \dot{z} = B + (x - C)z, \end{cases} \tag{S3}$$

where $A = 0.15$, $B = 0.2$. Firstly, we obtain the time series of different states by changing the parameter C. (*i.e.*,Rössler1: $C = 3.4$; Rössler2: $C = 4.4$; Rössler3: $C = 5.5$; Rössler4: $C = 5.9$.) The time series of one-period, two-period, four-period and eight-period Rössler system are obtained by numerical calculation, and the integration step $\Delta t = 0.02$. During the training phase, the parameter setting of the "calibrated" reservoir computing is $\eta = 1 \times 10^{-8}$, the average degree $D = 9.5$, the bias constant ξ=0.1, and the reservoir network nodes $N = 95$.

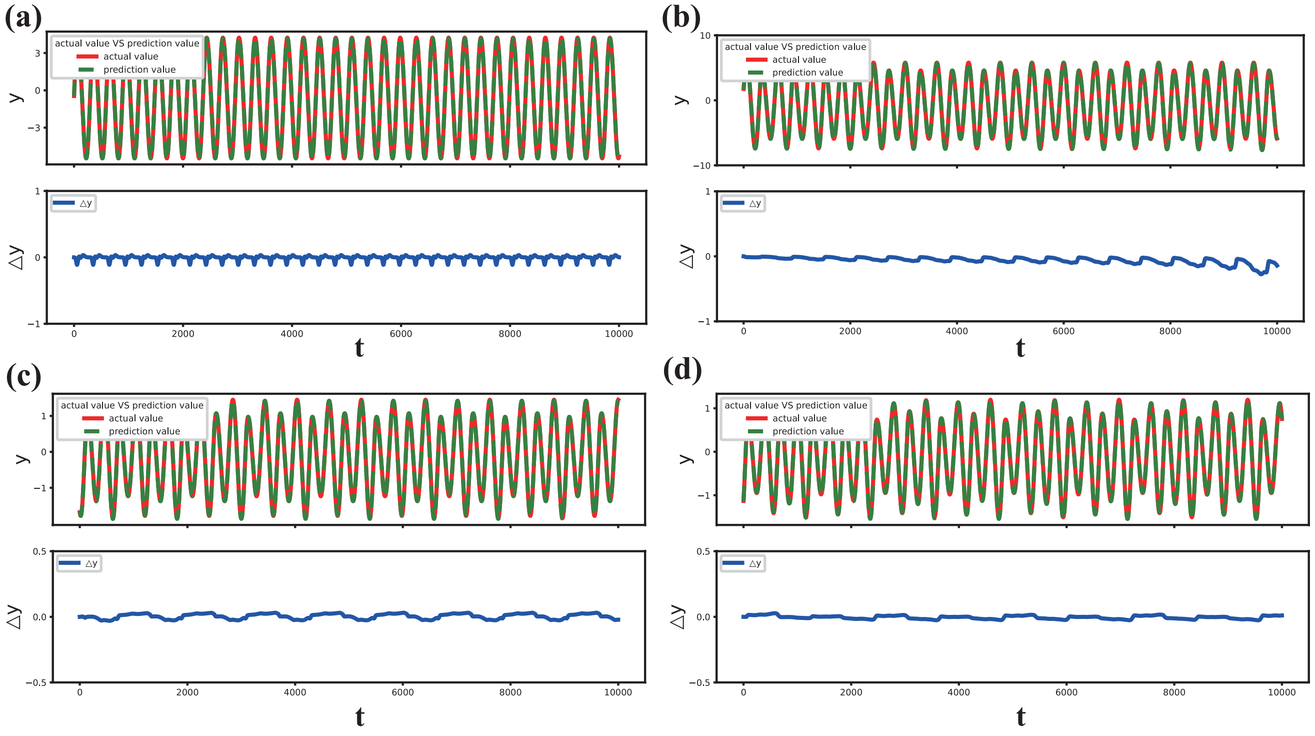

Figure S8. The time diagrams and the difference between reconstructed values and actual values of different periodic Rössler systems. (a)-(d) correspond to the $x - yz$ mode of one-period, two-period, four-period and eight-period of Rössler system respectively.

Table SVIII. Shortest training lengths of different Rössler systems in case 2($T_{test} = 10^4$, $RMSE$ <0.1)

Model style	$Min(T_{train})$	T_{test}	Model style	$Min(T_{train})$	T_{test}
Rössler1: $x - yz$	115	10000	Rössler2: $x - yz$	535	10000
Rössler1: $y - xz$	150	10000	Rössler2: $y - xz$	550	10000
Rössler1: $z - xy$	385	10000	Rössler2: $z - xy$	925	10000
Rössler3: $x - yz$	510	10000	Rössler4: $x - yz$	585	10000
Rössler3: $y - xz$	825	10000	Rössler4: $y - xz$	685	10000
Rössler3: $z - xy$	1945	10000	Rössler4: $z - xy$	2090	10000

Similarly, we select the shortest training length as the index of reconstruction effect when the reconstruction length is fixed as 10^4 and the $RMSE$ <0.1. The results of different Rössler systems are shown in Fig. S8 and Table SVIII. Note that, the hyperparameters are adjusted to get the shortest training length in each mode.

We can see that whether the states of Rössler is periodic or chaotic, the best reconstruction effect always is $x-yz$ mode. The results show there is a relationship between the efficiency of reconstruction and the equation structure. The variable x, which appears in both the equations $\dot{y}$, $\dot{z}$, performs best at reconstructing the variables y, z in all cases.

3 Reconstruction results of some classical dynamical systems

In this section, we test the "calibrated" reservoir computing model in more classical dynamical systems, where the parameters are all set as chaotic states. The corresponding equations of these systems are shown below:

Shimizu-Morioka(S-M)[2] system:

$$\begin{cases} \dot{x} = y \\ \dot{y} = (1-z)x - Ay \\ \dot{z} = x^2 - Bz, \end{cases} \tag{S4}$$

where, $A = 0.75, B = 0.45$.

Rucklidge(Ruck)[3] system:

$$\begin{cases} \dot{x} = -Ax + By - yz \\ \dot{y} = x \\ \dot{z} = -z + y^2, \end{cases} \tag{S5}$$

where, $A = 2, B = 6.7$.

Genesio-Tesi(G-T)[4] system:

$$\begin{cases} \dot{x} = y \\ \dot{y} = z \\ \dot{z} = -Ax - By - Cz + x^2, \end{cases} \tag{S6}$$

where, $A = 0.44, B = 1.1, C = 1$.

Wang-Sun(W-S)[5] system:

$$\begin{cases} \dot{x} = Ax + yz \\ \dot{y} = Bx + Cy - xz \\ \dot{z} = -z - xy, \end{cases} \tag{S7}$$

where, $A = 0.2, B = -0.01, C = -0.4$. For these systems, the parameter setting of the "calibrated" reservoir computing is $\eta = 1 \times 10^{-8}$, the average degree $D = 9.5$, the bias constant ξ=0.1, and the number of reservoir network nodes $N = 95$. The integration time step is $\Delta t = 0.02$. And the reconstruction results are shown in Fig. S9 and Table SIX.

All of these results indicate that the more interactions a variable has with other in dynamical equations, the better reconstruction of this variable is in reconstructing other. These systems, however, cannot completely confirm the relationship between the nonlinear terms and the reconstruction.

Table SIX. Shortest training lengths of some classical dynamical systems in case 2 ($RMSE$ <0.1)

Model style	$Min(T_{train})$	T_{test}	Model style	$Min(T_{train})$	T_{test}
S-M: $x-yz$	710	10000	Ruck: $x-yz$	615	10000
S-M: $y-xz$	760	10000	Ruck: $y-xz$	370	10000
S-M: $z-xy$	1690	10000	Ruck: $z-xy$	660	10000
G-T: $x-yz$	1470	10000	W-S: $x-yz$	2240	8000
G-T: $y-xz$	1000	10000	W-S: $y-xz$	2200	8000
G-T: $z-xy$	1475	10000	W-S: $z-xy$	2280	8000

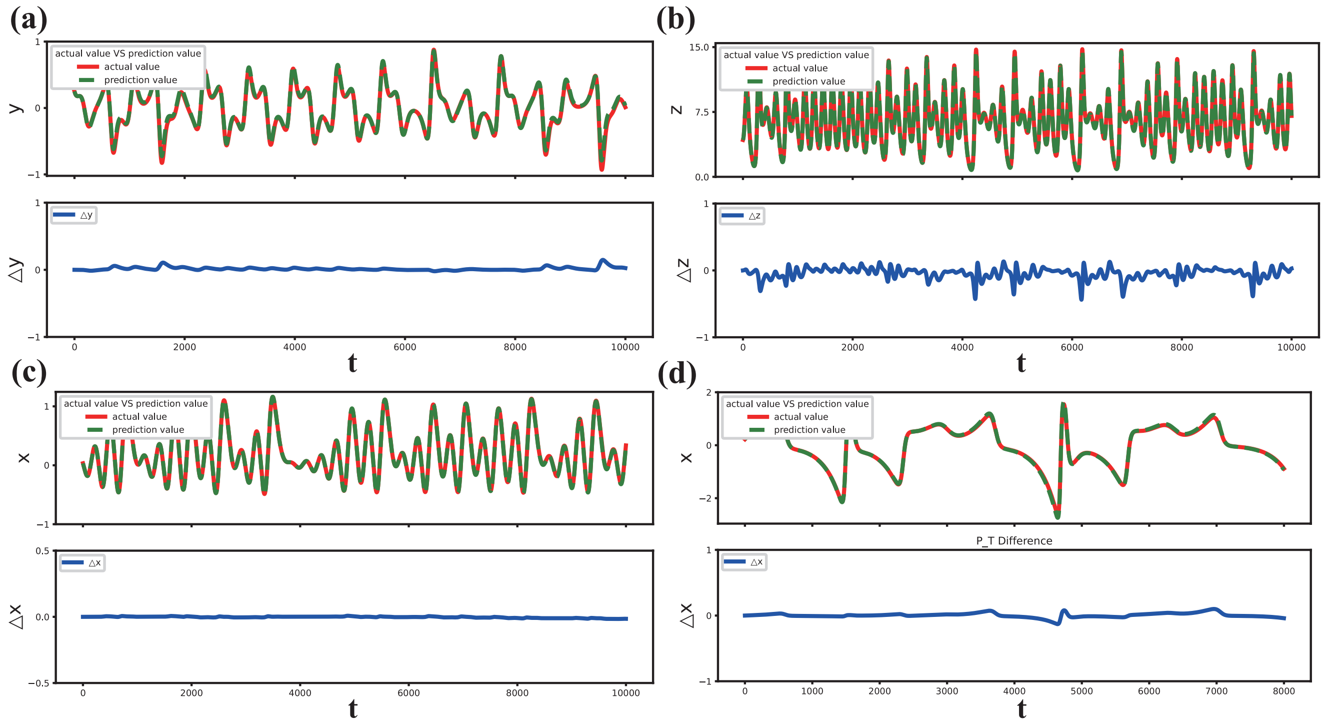

Figure S9. The time diagrams and the difference between reconstructed values and actual values of some classical dynamical systems. (a)-(d) correspond to the best mode of S-M system, Ruck system, G-T system, and W-S system, respectively.

4 Reconstruction results of "xy-yz" and "xz-yz" systems

In this section, we build two systems to verify the relation between the efficiency of reconstruction and nonlinear terms for the other two situations (when the system contains xy, yz and xz, yz), the math equations are as follows:

$$\begin{cases} \dot{x} = Ay + Byz \\ \dot{y} = C(y - z) \\ \dot{z} = D(xy + x) - Ez, \end{cases} \tag{S8}$$

where,$A = 14.1, B = 5.0, C = -18.8, D = -21.5, E = 2.0$.

$$\begin{cases} \dot{x} = yz - Ax \\ \dot{y} = Bz - xz + y \\ \dot{z} = C(2y - z) + Dy, \end{cases} \tag{S9}$$

where, $A = 3.17, B = 3.93, C = 8.04, D = 12$. During the training phase, the parameter setting of the "calibrated" reservoir computing is $\eta = 1 \times 10^{-8}$, the average degree $D = 9.5$, the bias constant ξ=0.1, and the number of reservoir network nodes $N = 95$. The integration time step is $\Delta t = 0.02$. The reconstruction results are shown in Fig. S10 and Table SX, we can see that the reconstruction of the $xz - y$ mode is better than the other modes ($yz - x$, $xy - z$) in the "xy-yz" system. It is conformed to our finding: the variable y is the repeated one, so it can be reconstructed to the best. Likewise, the best reconstruction in the "xz-yz" system is the $xy - z$ mode, due to the variable z being the repeated one in the nonlinear terms(xy, yz). All these results indicate that the efficiency of reconstruction of the system can play a certain role in revealing the dynamic features.

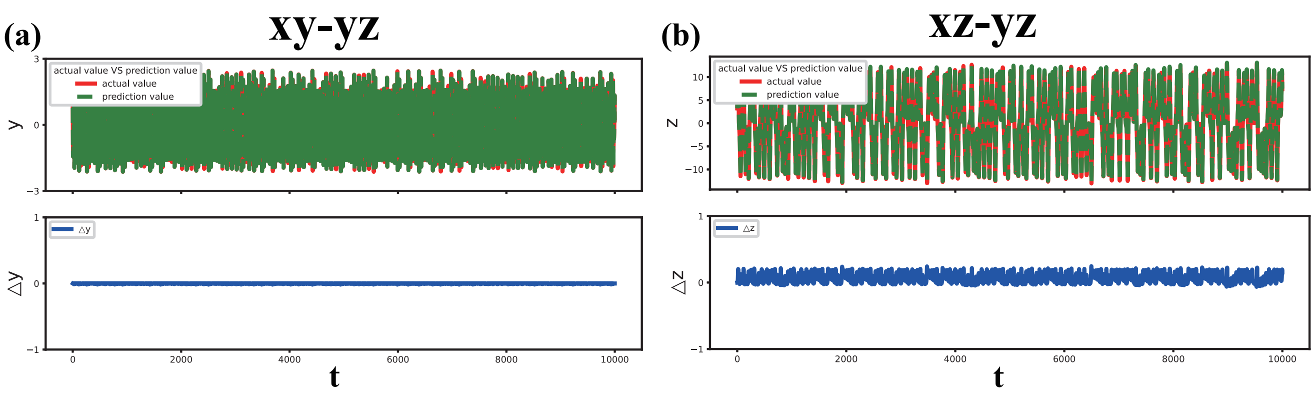

Figure S10. The time diagrams and the difference between reconstructed values and actual values of "xy-yz" and "xz-yz" systems. (a), (b) correspond to the best mode of "xy-yz" and "xz-yz" systems respectively.

Table SX. Shortest training lengths of Eq. (S8) and Eq. (S9) in case 1 (*RMSE* <0.1)

Model style	$Min(T_{train})$	T_{test}	Model style	$Min(T_{train})$	T_{test}
Eq. (S8): $yz-x$	40	10000	Eq. (S9): $yz-x$	135	10000
Eq. (S8): $xz-y$	30	10000	Eq. (S9): $xz-y$	180	10000
Eq. (S8): $xy-z$	50	10000	Eq. (S9): $xy-z$	25	10000

References

1. Rössler, O. E. An equation for continuous chaos. *Phys. Lett. A* **57**, 397–398 (1976).
2. Shimizu, T. & Morioka, N. On the bifurcation of a symmetric limit cycle to an asymmetric one in a simple model. *Phys. Lett. A* **76**, 201–204 (1980).
3. Rucklidge, A. M. Chaos in models of double convection. *J. Fluid Mech.* **237**, 209–229 (1992).
4. Genesio, R. & Tesi, A. Harmonic balance methods for the analysis of chaotic dynamics in nonlinear systems. *Automatica* **28**, 531–548 (1992).
5. Wang, Z., Sun, Y., van Wyk, B. J., Qi, G. & van Wyk, M. A. A 3-d four-wing attractor and its analysis. *Braz. J. Phys.* **39**, 547–553 (2009).
